# Human *α-L-fucosidase-1* attenuates the invasive properties of thyroid cancer

**DOI:** 10.18632/oncotarget.15635

**Published:** 2017-02-23

**Authors:** Giancarlo Vecchio, Alessia Parascandolo, Chiara Allocca, Clara Ugolini, Fulvio Basolo, Marco Moracci, Andrea Strazzulli, Beatrice Cobucci-Ponzano, Mikko O. Laukkanen, Maria Domenica Castellone, Nobuo Tsuchida

**Affiliations:** ^1^ Dipartimento di Medicina Molecolare e Biotecnologie Mediche, Università di Napoli Federico II, Via Sergio Pansini, 5, Naples, Italy; ^2^ Istituto Superiore di Oncologia, Via Sergio Pansini, 5, Naples and Via Balbi 5, Genoa, Italy; ^3^ IRCCS SDN, Naples, Italy; ^4^ Dipartimento di Medicina di Laboratorio, Azienda Ospedaliero-Universitaria Pisana, Pisa, Italy; ^5^ Dipartimento di Patologia Chirurgica, Medica, Molecolare e dell'Area Critica, University of Pisa, Italy; ^6^ Institute of Biosciences and Bioresources (IBBR), National Research Council of Italy (CNR), Via P. Castellino, 111, Naples, Italy; ^7^ Department of Biology, University of Naples “Federico II”, Complesso Universitario di Monte S. Angelo, Naples, Italy; ^8^ Istituto di Endocrinologia ed Oncologia Sperimentale “G. Salvatore” (IEOS), CNR, Naples, Italy; ^9^ Graduate School of Medical and Dental Sciences Tokyo Medical and Dental University, Tokyo, Japan

**Keywords:** glycosylation, fucosidase, thyroid carcinoma, metastasis

## Abstract

Glycans containing α-L-fucose participate in diverse interactions between cells and extracellular matrix. High glycan expression on cell surface is often associated with neoplastic progression. The lysosomal exoenzyme, α-L-fucosidase-1 (FUCA-1) removes fucose residues from glycans. The *FUCA-1* gene is down-regulated in highly aggressive and metastatic human tumors. However, the role of FUCA-1 in tumor progression remains unclear. It is speculated that its inactivation perturbs glycosylation of proteins involved in cell adhesion and promotes cancer. FUCA-1 expression of various thyroid normal and cancer tissues assayed by immunohistochemical (IHC) staining was high in normal thyroids and papillary thyroid carcinomas (PTC), whereas it progressively decreased in poorly differentiated, metastatic and anaplastic thyroid carcinomas (ATC). *FUCA-1* mRNA expression from tissue samples and cell lines and protein expression levels and enzyme activity in thyroid cancer cell lines paralleled those of IHC staining. Furthermore, ATC-derived 8505C cells adhesion to human E-selectin and HUVEC cells was inhibited by bovine α-L-fucosidase or Lewis antigens, thus pointing to an essential role of fucose residues in the adhesive phenotype of this cancer cell line. Finally, 8505C cells transfected with a *FUCA-1* containing plasmid displayed a less invasive phenotype versus the parental 8505C. These results demonstrate that FUCA-1 is down-regulated in ATC compared to PTC and normal thyroid tissues and cell lines. As shown for other human cancers, the down-regulation of FUCA-1 correlates with increased aggressiveness of the cancer type. This is the first report indicating that the down-regulation of FUCA-1 is related to the increased aggressiveness of thyroid cancer.

## INTRODUCTION

Glycosylation is an important physiological and pathological process in which oligosaccharides are added to proteins or lipids. Alterations of glycosylation patterns on cell surface proteins are common features of malignant neoplastic transformation, and aberrant glycosylation is involved in key steps of neoplastic transformation, including tumor invasion and metastases [1–6]. α-L-fucose, a monosaccharide component of glycosylated protein and lipid, is involved in interactions of various types of cell-cell, and cell-ECM (extracellular matrices), including adhesion to the vessel endothelium [7, 8]. Fucose can be added to existing glycans to yield more complex glycans. This process leads to the synthesis of small carbohydrates such as Lewis antigens. Fucose frequently exists as a terminal modification of glycan structures; however glycosyltransferase activities capable of adding sugar directly to fucose have been identified. Fucosylated glycans are synthesized by fucosyltransferases; thirteen fucosyltransferase genes have ben identified in the human genome [9]. High α-L-fucose expression has been reported in various solid tumors [10–14] including thyroid carcinomas [13] and its levels often directly correlate with neoplastic progression [10, 11]. Moreover, elevated fucose levels are preferentially expressed in metastatic foci versus primary tumors [10–12]. Core fucosylation (*N-linked* oligosaccharides in glycoproteins via α-1,6-linkage to fucose), has been shown to be altered in prostate cancer [15, 16] and sera of patients [17]. Yuan and colleagues showed high levels of α-L-fucose on the surface of human breast cancer cells [18].

Thyroid tumors, whose incidence appears to be increased in recent years (even though higher sensitivities of detection techniques could contribute to such an increase) [19, 20], are the most frequent neoplasias of the endocrine system. Thyroid malignant tumors are classified in five histological types: papillary (PTC) and follicular (FTC), which are differentiated thyroid carcinomas, poorly differentiated (PDTC), anaplastic or undifferentiated (ATC) and medullary (MTC).

Although differentiated thyroid cancers have a generally favorable prognosis, patients affected by tumors with distant metastases display elevated morbidity and mortality. The presence of distant metastases at diagnosis is, in fact, the most negative prognostic sign for differentiated thyroid tumors. Mortality for metastatic differentiated tumors is about 50% at 10 years [21]. ATCs are the most aggressive thyroid tumors with a mortality rate among the highest of all cancers and with a mean survival at diagnosis of 6 months [22]. Up to date the only efficient therapy for metastatic differentiated thyroid carcinomas is that consisting in the administration of radioactive iodide. There are no efficient therapies for patients affected by metastatic thyroid carcinomas that are not responsive to this type of therapy. Neither chemotherapy nor radiotherapy is capable of prolonging survival of patients affected by ATC with distant metastases [22]. These data emphasize the need to identify new molecular markers able to distinguish thyroid differentiated cancers with good from those with bad prognosis. These data also emphasize the need to treat patients affected by thyroid cancer before the appearance of distant metastases.

We report here a significantly lower expression of FUCA-1 in anaplastic thyroid tumors when compared with that of papillary thyroid carcinomas. Furthermore, an ATC-derived cell line showed an invasive behavior, which was attenuated after transfection with *FUCA-1* DNA. On the contrary, silencing of *FUCA-1* in the papillary thyroid cancer TPC-1 cell line, that expressed high levels of the enzyme, increased its invasive behavior *in vitro*. The results, taken together with the published observations, suggest that high FUCA-1 expression could decrease the abundance of fucose-containing glycans on the surface of cancer cells, and thereby attenuating tumor cell invasion. Therefore, our data indicate for the first time that the decrease of FUCA-1 levels could be regarded as a potential marker for aggressive thyroid cancer cell types.

## RESULTS

### Fuca-1 expression is reduced in anaplastic thyroid cancer tissue samples and cell lines

**Figure 1:**
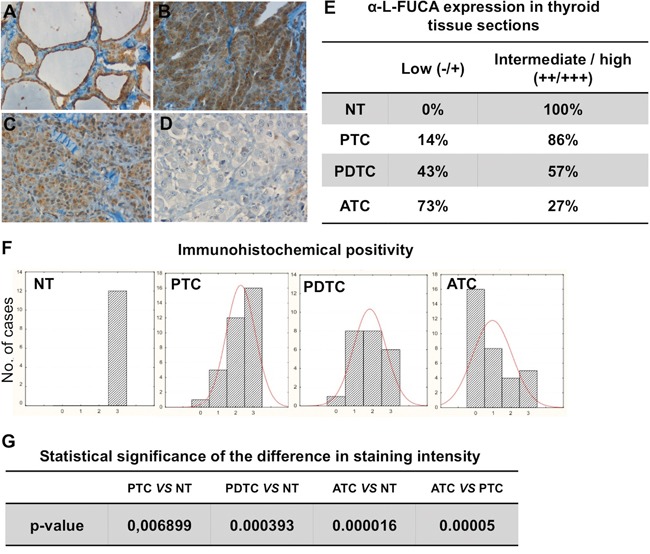
Immunohistochemical staining of tissue sections from normal thyroid (NT) A., from one sample of a PTC B., one sample of PDTC C. and one sample of ATC D. with the anti-α-L-FUCA-1 antibody The staining is clearly visible only in the normal thyroid tissue and in the PTC samples, whereas it is very low in the PDTC and absent in the ATC samples. (Magnification 20x). Degrees of staining intensity (from 0 to +++) of all normal, PTC, PDTC and ATC samples analyzed by immunohistochemistry with the anti-α-L-FUCA-1 antibody **E, F**. Statistical significances of the difference in immunohistochemical intensity between normal thyroids and different thyroid cancer types **G**.

Expression levels of FUCA-1 in less aggressive thyroid cancers (PTC), in poorly differentiated thyroid carcinomas (PDTC), which represent an histological subtype with intermediate clinical behavior between differentiated and undifferentiated or anaplastic carcinomas, and in highly malignant invasive and metastatic anaplastic thyroid cancers (ATC) were compared with those of normal thyroid tissues (NT). A set of archived histological sections was examined by immunohistochemistry (IHC) with a commercially available antibody against human FUCA-1. In particular, we have examined 33 PTCs, 26 PDTCs, 33 ATCs, and 12 tissue samples obtained from patients operated for non-thyroidal tumor pathologies. Figure 1 shows the immunostaining, with the FUCA-1 antibody, of positive normal thyroid tissue (Figure 1A) compared with those of positive PTC (Figure 1B), the partially negative PDTC (Figure 1C) and the completely negative ATC (Figure 1D). FUCA-1 expression levels were classified into high (+++), intermediate (++) or low (+/0). High levels of expression were observed in all normal thyroid tissues and in 86% of PTCs, in 57% of PDTCs and in a much lower proportion of ATCs (27%), whereas low levels of expression were observed in 14% of PTCs, in 43% of PDTCs and in 73% of ATCs (Figure 1E).

**Figure 2:**
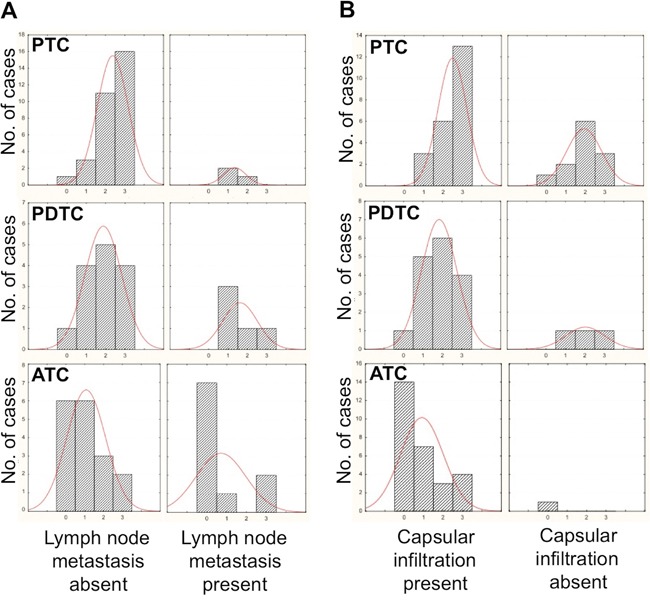
Correlation between staining intensity with the anti-α-L-FUCA-1 antibody and presence or absence of lymph-nodal metastases in all PTC, PDTC and ATC samples analyzed **A**. Correlation between staining intensity with the anti-α-L-FUCA-1 antibody and presence or absence of capsular infiltration of all PTC, PDTC and ATC samples analyzed **B**.

Histograms for sample numbers of each of expression level (0/+, ++, or +++) were made for each thyroid tumor type and compared (Figure 1F). All normal samples displayed the highest level of positivity (+++), while the number of samples with the highest level (+++) decreased from PTC, through the PDTC to the ATC samples. Further, the expression levels of PTC samples were more scattered among the tested tumor types, while all normal samples showed uniformly high levels (+++). The analysis of these results showed that differences between normal thyroids and PDTC or ATC samples were statistically significant. The difference in positivity between PTCs and normal samples, although still significant, was characterized by a higher p value, while the difference in positivity between PTC and ATC samples was highly significant (Figure 1G). A statistical analysis was further performed on the correlation between presence or absence of lymph-nodal metastases at diagnosis of the PTC, PDTC and ATC patients and expression levels of FUCA-1 in the tumor samples (Figure 2A). The results demonstrate that LNM- PTC, PDTC and ATC patients expressed higher levels of FUCA-1, compared to LNM+ PTC, PDTC and ATC patients.

A similar inverse correlation for all PTC, PDTC and ATC samples analyzed, between capsular invasion and expression levels of FUCA-1 was evident, although only for the PTC samples the correlation was significant, due to the low number of PDTCs and ATCs not showing capsular invasion (see Figure 2B).

**Figure 3:**
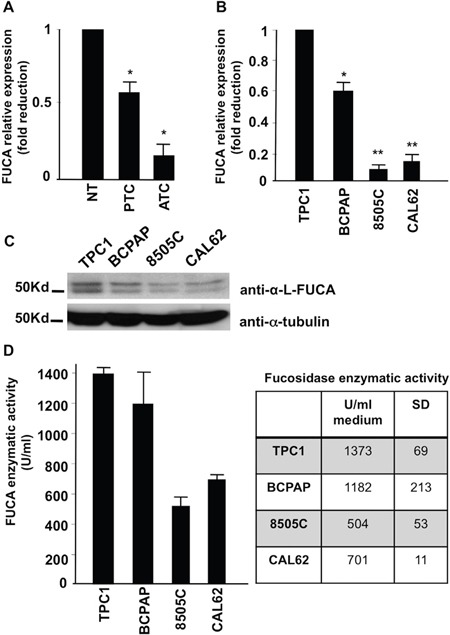
Expression of a-L-FUCA-1 mRNA of normal thyroid tissues (NT) (5), papillary (PTC) (14) and anaplastic (ATC) (8) thyroid cancer biopsies (*=p<0.05) **A**. Real time PCR of the mRNA for a-L-FUCA-1 extracted from two papillary (TPC-1 and BCPAP) and two anaplastic (CAL-62, 8505C) thyroid cancer cell lines, normalized for the TPC-1 cell line (*=p<0.05; **= p<0.01) **B**. Western blot analysis of cell lines derived from human thyroid papillary cancer (TPC-1 and BCPAP), anaplastic thyroid cancer (8505C and CAL62). A doublet protein band, specific for FUCA-1, is present at high levels in the TPC-1 and BCPAP cell lines. The doublet band is present at low levels in the 8505 C cell line **C**. a-L-FUCA-1 enzymatic activity of cell extracts from TPC-1, BCPAP, 8505C, and CAL62 cell lines, measured at 37°C, pH=5.5 with 500 μM of 4-Methyl-umbellyferyl-a-L-fucopyranoside (4MU) substrate **D**.

To confirm the IHC results, we analyzed by Real Time PCR the *FUCA-1* mRNA expression on 5 thyroid tissue samples obtained from 5 different patients from whom thyroids were removed for benign thyroid diseases (NT), 8 biopsies from patients with ATC and 14 patients with PTC. The average mRNA fold reductions observed were 0.56 for PTCs and 0.20 for ATCs. The differences between normal and papillary, normal and anaplastic and papillary and anaplastic thyroid biopsies were statistically significant (p<0.05), thus confirming that FUCA-1 expression levels were more than twice in PTCs compared with ATCs also by measuring the *FUCA-1* mRNA levels (Figure 3A).

To test whether FUCA-1 expression is higher in more differentiated thyroid cancers, when compared with the more aggressive, undifferentiated thyroid cancers, we analyzed also human thyroid cell lines derived from patients with PTC or ATC. In particular, we examined the cell lines designated TPC-1 and BCPAP (papillary) and 8505C and CAL62 (anaplastic). *FUCA-1* gene expression tested by Real Time PCR showed that the anaplastic thyroid cancer cell lines analyzed displayed lower levels of the *FUCA-1* mRNA, compared with the two papillary cancer cell lines analyzed (TPC-1 and BCPAP) (see Figure 3B).

We next examined the FUCA-1-specific proteins in thyroid cancer cell lines (TPC-1 and BCPAP of PTC and 8505C and CAL62 of ATC). A doublet band with differential glycosylation, probably resulted from post-translational modification of the protein (although additional explanations for the doublet band cannot be excluded) was specifically recognized by Western blot with the anti-FUCA-1 antibody utilized in our experiments. Two nonidentical subunits have been reported for human semen [23], human liver [24], and an airway cell line [25]. Judging from the intensities of the doublet band, FUCA-1 was down-regulated in the ATC cell lines compared with the PTC cell lines (Figure 3C), thus confirming the observation obtained by IHC and PCR analysis on tissue samples.

#### FUCA-1 enzymatic activity was reduced in ATC cells

Cell extracts from two papillary (TPC-1 and BCPAP) and two anaplastic (8505C and CAL-62) cancer cell lines were assayed for FUCA-1 enzymatic activity, using 4-methyl-umbellyferyl-α-L-fucopyranoside (4-MU) as a substrate. Figure 3D shows that the average activities of FUCA-1 were highest (1373 U/ml) in the TPC-1 cell line, intermediate (1182 U/ml) in the BCPAP cell line and minimal (701 and 504 U/ml) in the CAL62 and 8505C lines, respectively, thus confirming the results of IHC and Real Time PCR with thyroid cancer biopsies and those of Western blotting with thyroid cell lines. Therefore, it is concluded that the enzyme activity was down-regulated in ATC with respect to PTC and to normal thyroid tissue, paralleling the reduced expression of the protein and mRNA.

### Adhesion of anaplastic 8505C cells to a HUVEC monolayer

#### Extracellular treatment with bovine α-L-fucosidase enzyme reduced the adhesion

**Figure 4:**
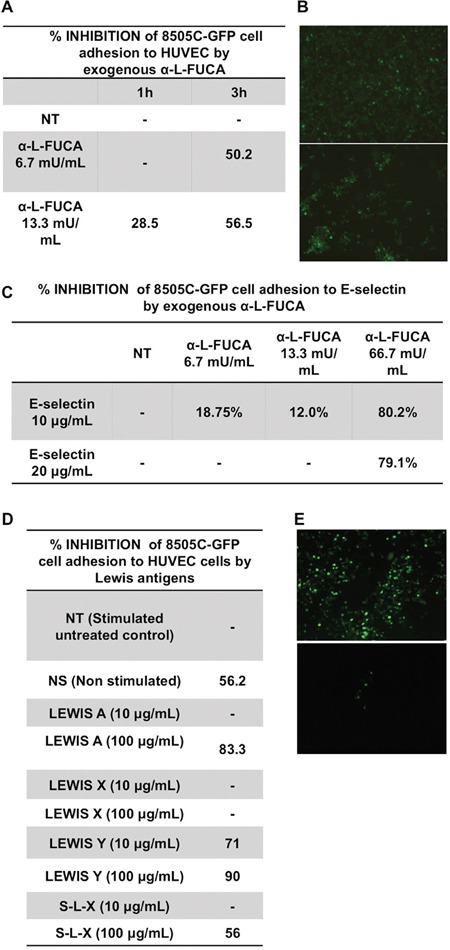
Inhibition of the adhesion to HUVEC cells of 8505C anaplastic thyroid cancer cells by increasing concentrations of bovine α-L-fucosidase **A, B**. Microphotographs of a layer of 8505C-GFP labeled cells adhering to a HUVEC monolayer after incubation of untreated cells (NT) for 3 hours at 37° (upper panel) or after incubation of the 8505C-GFP labeled cells treated for 3 hours at 37° with a final concentration of 13.3 mUnits/ml α-L- bovine fucosidase (α-L- FUCA) (lower panel). The microphotographs were taken after washing the nonspecifically adhered cells. (See Materials and Methods). **C**. Inhibition of the adhesion of 8505C cells to E-selectin by increasing concentrations of bovine α-L-fucosidase for increasing incubation times (1h and 3 h). **D**. Inhibition of the adhesion of 8505C-GFP cells to the HUVEC monolayer by increasing concentrations of Lewis antigens. **E**. Microphotographs of a layer of 8505C-GFP labeled cells adhering to a HUVEC monolayer after incubation of untreated cells for 3 hours at 37° (left panel) or after incubation of the 8505C-GFP labeled cells treated for 3 hours at 37° with a final concentration 100 μg/ml Lewis Y antigen (right panel). The microphotographs were taken after washing the non specifically adhered cells (see Materials and Methods).

The highly aggressive, metastatic, and anaplastic thyroid cancer cells, 8505C were found capable to adhere to a monolayer of stimulated HUVEC cells, while a strong adhesion reduction was observed on un-stimulated cells, thus indicating the specificity of the adhesion process. In order to examine the role of fucose residues of the cell surface glycans in the adhesion, the 8505C cells were fluorescently labeled with GFP (8505C-GFP) and treated with increasing concentrations of bovine α-L-fucosidase. The adherent cells were counted under a fluorescent microscope (see Materials and Methods for details) (Figure 4A). As shown in Figure 4A, the pre-incubation of 8505C cells with bovine α-L-fucosidase suppressed the adhesion to the HUVEC monolayer. No significant loss of viability of 8505C cells was detected up to 3 hours with the highest enzyme concentration (not shown). This result suggests that the bovine FUCA-1 treatment attenuated the binding of 8505C cells to endothelial cells. Figure 4B shows the microphotographs of untreated 8505C-GFP cells (upper panel) and bovine α-L-fucosidase- treated cells (lower panel) (final concentration of 13.3 mUnits/ml), which adhered to the HUVEC monolayer. The number of adherent cells was reduced approximately three folds by the enzyme treatment.

#### Extracellular treatment with bovine Fuca-1 reduced the binding to E-selectin

Adhesion of cancer cells to endothelial cells has been reported to occur via binding of the glycan moieties of glycoproteins and glycolipids to their natural main receptors such as E- and P-selectins. 8505C-GFP cells were examined also for the inhibition of adherence to E-selectin after extracellular treatment with increasing concentrations of bovine α-L-fucosidase. Figure 4C shows that the adhesion of 8505C cells to E-selectin was inhibited by about 80% with the bovine enzyme at a high concentration (66.7 mU/ml), thus suggesting that the removal of fucose moieties from surface glycans of 8505C cells reduced their adhesion to the natural E-selectin receptor on the endothelial cells.

#### Lewis antigens interfered with the adhesion of 8505C cells to the HUVEC cell layer

The adhesion of cancer cells to the endothelial cells has been reported to be mediated by the fucose containing-Lewis antigens present on the cell surface glycans. These antigens have been considered as the natural ligands in the binding and adhesion processes to E-selectin [26, 27]. Therefore, we examined whether the Lewis antigens interfere with the adhesion of 8505C-GFP cells to a HUVEC cell layer in the presence of increasing concentrations of four different types of Lewis antigens: [1] Lewis A, so-called type 1 Lewis antigen, a trisaccharide containing one fucose residue, linked with a α 1-4 link to GlcNAcβ, [2] Lewis X, a trisaccharide containing one fucose residue linked in a α 1-3 linkage to GlcNAcβ, [3] Lewis Y, a tetrasaccharide containing two fucose residues, one bound as in Lewis X and the other one linked as an α 1-2 link to Galβ, and [4] Sialyl-Lewis X (S-L-X), a tetrasaccharide containing one fucose residue bound to GlcNAc with an α 1-3 linkage. Lewis X, Lewis Y and Sialyl Lewis X are also known as type 2 Lewis antigens. Lewis antigens were used to test whether they inhibited the binding of the Lewis antigens naturally present on surface glycoproteins and glycolipids to their E-and P-selectin ligands, by displacement, thereby interfering with the binding of cancer cells to the HUVEC cell layer. The results in Figure 4D show that 100 μg/ml of Lewis A, or Lewis Y, inhibited the adhesion by 80-90%, whereas the same concentrations of Sialyl Lewis X or Lewis X inhibited the adhesion by 44% or 0% respectively. No significant loss of viability of 8505C cells was detected in the presence of the highest concentrations of each Lewis antigen used (not shown). Figure 4E shows the adhesion of 8505C-GFP cells which were not treated (left panel) or treated with Lewis Y (final concentration 100μg/ml, equivalent to 149.8 μM) (right panel). The number of adherent cells counted in the control sample was 467 against 18 adherent cells counted in the Lewis Y-treated cells. Thus, these results indicate a possible role of the epitopes of Lewis A, Y and Sialyl-Lewis X antigens in the adhesion of 8505C cells to endothelial cells.

### Transfection of 8505C cells with FUCA-1 (8505C/FUCA-1) attenuated the malignant behavior of anaplastic thyroid cells

#### Establishment of 8505C/FUCA-1 cells

**Figure 5:**
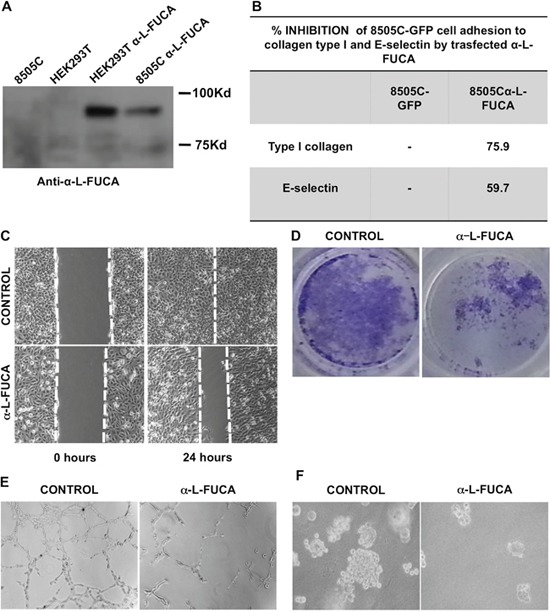
A. Western blot analysis of protein extracts obtained from 8505C (lane 1), HEK293T cells (lane 2), HEK293T cells transfected with the α-L-FUCA-1 plasmid (lane 3), 8505C cells transfected with the α-L-FUCA-1 plasmid (lane 4) The membrane was probed with the anti-FUCA-1 antibody (Proteintech, see Materials and Methods). The 85 kDa recombinant protein band is clearly visible with both the HEK293T and 8505C cells transfected with the α-L-FUCA-1 plasmid. **B**. Inhibition of 8505C cells adhesion to collagen type I and to E-selectin by endogenous α-L-FUCA-1. **C**. Parental (left panels) and transfected (right panels) 8505 C cells at time 0 after the wound and at 24 h after the wound. **D**. Either parental 8505C or 8505C/FUCA-1 cells (10^5^ cells each) were plated at time 0 in Matrigel containing canisters and the migration was measured by staining the migrated cells after 48 hours and photographed (see Materials and Methods). **E**. Either parental 8505C or 8505C/L-FUCA-1 cells (10^5^ cells) were plated at 0 time in Matrigel 3 D containing canisters and the migration was observed after 24 hours under an inverted microscope and photographed (see Materials and Methods). **F**. Either parental 8505C or 8505C/L-FUCA-1 cells were plated in duplicates onto an agar layer in 60 mm plates (see Materials and Methods) and grown for 15 days after plating. Negative control was represented by NIH3T3 cells, positive control by NIH3T3 cells transformed with the *RET MEN 2A* oncogene (5028 cells) (not shown). The colonies were photographed at day 15 after plating under an inverted microscope.

We established a mass population of 8505C cells stably carrying the human *FUCA-1* with a GFP-tag (8505C/FUCA-1). An 85Kd GFP tagged FUCA-1 protein was detected by Western blot with either anti-FUCA-1 or with an anti- GFP antibody (Figure 5A). This band was not present in non-transfected 8505C. This recombinant protein was fully functional, as assayed by enzymatic activity (data not shown). HEK293T cells were used as a transfection control (HEK293T/FUCA-1) (Figure 5A). Interestingly, we have observed that expression of exogenous FUCA-1 protein decreased upon *in vitro* passaging, suggesting that the same mechanism acting by reducing the endogenous protein, may function also on the exogenous FUCA-1 expression. In the following sections, the biological properties of low passages 8505C/FUCA-1 cells, still expressing the FUCA-1 protein, have been compared with those of parental 8505C cells.

#### Adhesion of 8505C/FUCA-1 cells to E-selectin or collagen

The adhesive properties of 8505C/FUCA1 cells were tested by assaying the ability of these cells to adhere to E-selectin or collagen-coated microtiter plates. Figure 5B shows that the adhesion to E-selectin was reduced in the 8505C/FUCA-1 cells when compared to that of 8505C cells transfected with a plasmid containing only GFP (8505C/GFP), suggesting that the protein made by *FUCA-1* transfected cells was capable of inhibiting the adhesion in the same manner as the cells treated with extracellular bovine α-L-fucosidase enzyme. Similar results were obtained when adhesion to collagen was measured (Figure 5B).

#### Wound healing test

8505C/FUCA-1 cells were tested for their wound healing (migration activity), and compared with the parental 8505C cells (Figure 5C). Left panels show the results of the wound healing experiment at 0 time (immediately after the wound), observed for the parental and transfected cell line, respectively, whereas right panels show the results observed 24 hours after the wound. The results indicate that the healing was completed in the parental cells but not in the 8505C/FUCA-1 cells. These results suggest that the expression at high levels of FUCA-1 attenuated the migration properties of the transfected cells when compared to the parental cells that expressed low levels of the enzyme.

In order to exclude that the slower migration and the longer wound healing time of the transfected versus the parental cell line was due to a slower growth rate of the transfected cells, we compared growth curves of the two cell lines. The two cell lines grew almost identically (Supplementary Figure 1).

#### Matrigel invasion assay

Figure 5D shows the results of a Matrigel assay for the parental 8505C and 8505C/FUCA-1 cells respectively. The cell number penetrated into the lower chamber through the Matrigel, representing invading cells, was much lower for 8505C/FUCA-1 than that of the parental cells, thus confirming that the levels of FUCA-1 expression were inversely related to the invasive properties of the cell lines, being higher in the cells expressing low levels of the enzyme.

We further performed Matrigel 3 D experiments and have confirmed that also with this type of matrix the levels of migration and chain formation of the two cell lines were strikingly different, being higher with the parental than with the transfected cells (see Figure 5E).

#### Growth in Soft agar

In keeping with the above results, experimens performed by growing parental and transfected cells in soft agar demonstrated that the 8505C/*FUCA-1* cells formed a lower number and smaller colonies in soft agar than the parental 8505C cells (Figure 5F).

### Silencing of FUCA-1 in the TPC-1 papillary thyroid cancer cell line

#### Analysis by real time PCR

The correlation between low FUCA-1 expression and invasive properties of the thyroid cancer cells was confirmed by silencing *FUCA-1* in the less aggressive papillary TPC-1 cells, which constitutively expressed high levels of FUCA-1. sh-RNAs directed against various portions of *FUCA-1* were used to obtain several mass populations and clones stably silenced for *FUCA-1*. The Real Time PCR experiment (qRT-PCR), shown in Supplementary Figure 2, demonstrated a significant decrease of *FUCA1* mRNA levels in all silenced clones (sh1FUCACl1, sh1FUCACl2, sh1FUCACl3, sh3FUCACl2 and in GFP-sorted sh1FUCAcl1S and sh1FUCAcl3S), when compared to the control sh-scrambled TPC1 cells (shSCRcl1) (Supplementary Figure 2).

#### Biological properties of the *FUCA-1*-silenced TPC-1 cells

Two *FUCA-1*-silenced TPC-1 cell clones, FUCAsh1cl 1 and FUCAsh1cl3 were compared to the sh-scrambled transfected TPC1 shSCRcl1 cells for their adhesion capacity to a collagen coated microtiter plate (Supplementary Figure 3A). The adhesion of *FUCA-1*-silenced TPC-1 cells to collagen was increased for both clones (123% and 160%) with respect to the control sh-scrambled TPC-1 cells, suggesting that obtained silencing of *FUCA-1* enhanced the adhesion to the collagen matrix. Thus, the results obteined strengthened the possible role of *FUCA-1* in inhibiting the attachment to the extracellular matrix, which is the first step in the invasion process of malignant cells.

The *FUCA-1-*silenced TPC-1 clones were also analyzed for their invasive properties by both wound healing and Matrigel penetration. As shown in Supplementary Figure 3B, 3C, 3D, and 3E, the wound was totally closed by 36 hours after the wound in the sh-1-FUCA-Cl3 cells (Supplementary Figure 3E), while it was not yet completely repaired in the sh-scrambled TPC-1 cell population (Supplementary Figure 3C).

The capacity of the *FUCA-1*-silenced TPC-1 cells to penetrate Matrigel was also assayed (Supplementary Figure 3F and 3G). The sh-1-FUCA-Cl3 gained a much greater degree of penetration through Matrigel (Supplementary Figure 3G). while the sh-scrambled TPC-1 cell population showed a small degree of penetration (Supplementary Figure 3F). These results together indicate that the silencing of the *FUCA-1* in TPC-1, lead to a possible increase in the fucosylation of membrane glycoproteins, and thereby conferred more invasive properties to TPC-1.

### Differential gene expression profiling between parental and *FUCA-1*-transfected 8505C cells

We analyzed the differential gene expression profiling by comparing the mRNAs extracted from parental and *FUCA-1*-transfected 8505C cell lines, in order to find the possible differences in gene expression induced by *FUCA-1*. The analysis was performed by using a Qiagen Human Gene Expression microarray, containing oligonucleotide probes for the whole human genome, hybridized with cDNAs from quadruplicate samples of the two different cell lines (described above) labeled with two different fluorophores. Data obtained from the hybridization was analyzed to determine the statistically significant genes differentially expressed in the presence of FUCA-1. Supplementary Table 1 shows the genes down-regulated in 8505C/*FUCA-1* cells, which were grouped into [1] adhesion phenomena (ADAM proteins), [2] migration (chemokine receptor CXCR4, Rho GEFs, Rhotekin, TRIO, and Rap GEFs), [3] angiogenesis (VEGFA), [4] cell growth (myc, c-Kit, ras, cyclins, polo-like kinases, aurora kinases, FGFR, Insulin receptor, and SLITRK4), and [5] cell stemness, differentiation and epithelial/mesenchymal transition (EMT) (Wnt/β-catenin, Hedgehog, Yap/Taz, and Notch). Genes also down-regulated include those for proteins involved in the signaling of MAP kinases, of FAK (Focal Adhesion Kinase), of PKA and PKC. It is noteworthy that genes coding for the Rho guanine nucleotide exchange factors (GEFs such as ARHGEF5, FGD2, ARHGEF35, FGD4, ARHGEF37, ARHGEF40, and NGEF) were also down regulated. On the contrary, Rho GTPase activating proteins (GAPs) such as ARHGAP44, ARHGAP6, ARHGAP42, and ARHGAP9 were up-regulated in the presence of FUCA1 (Supplementary Table 2).

## DISCUSSION

In the present study, we demonstrated that, in thyroid tumors, FUCA-1 expression, as analyzed by immunohistochemistry, is related with the degree of differentiation of thyroid cancers: the levels in differentiated papillary thyroid cancers examined (PTCs), were relatively high, close to those of normal thyroid tissues. On the contrary, FUCA-1 levels were low in poorly differentiated (PDTCs) and lowest in anaplastic (ATCs) thyroid cancers, respectively. Furthermore, its expression was inversely correlated with the lymph-node involvement and with the capsular invasion, for all the subtypes of thyroid cancers examined. Similar conclusions were drawn from the results of FUCA-1 mRNA and protein expression in fresh biopsies of PTCs and ATCs, as well as in cell lines established from these tumors. In the latter ones a similar correlation was found also by examining the fucosidase enzyme activity.

Down-regulation of FUCA1 has been consistently associated with cancer progression. A meta-analysis of multiple microarray datasets obtained from 18 publicly available gene expression datasets in the Oncomine database (https://www.oncomine.org), comparing distant metastases to primary tumors in various solid tumors, revealed a common genetic signature of metastasis in solid tumors. FUCA1 gene was among the genes which were found to be down-regulated in 4 different datasets [28]. Lower FUCA-1 expression was associated with recurrences and lower cancer specific survival in breast cancer patients [29]. A decreased expression of the *FUCA1* gene was also found in human colorectal carcinomas compared to normal mucosa and further a gradual decrease in FUCA-1 expression was observed with progression of the disease from early to advanced stages [30]. A decrease of the α-L-fucosidase activities in the tumor *vs* normal mucosa was correlated with recurrence in more than 52% of a cohort of 123 colorectal carcinoma patients [31]. Furthermore, in a study of microarray analysis in which the gene expression patterns of neuroblastomas (NB), characterized by highly probable spontaneous regression, was compared with stage 4 NB, with poor prognosis, revealed a set of 19 discriminatory genes that may play a significant role in the natural progression of the disease. *FUCA-1* was among the 19 genes displaying a 5 folds down-regulation change in the set of poor prognosis patients [32].

On the other hand, higher FUCA-1 expression levels were correlated with a favorable progression free survival (PFS) in a cohort of breast cancer patients [33]. Higher expression was also considered as a good independent prognostic factor of tumor recurrence in colorectal carcinoma patients [31]. Thus, expression levels of FUCA-1 may predict the prognosis of mammary tumors, CRC, neuroblastomas and thyroid tumors.

The differences in FUCA-1 expression found by us between PTCs and ATCs have been confirmed by examining a panel of thyroid cancer cell lines derived from different PTCs and ATCs. Most ATC cell lines analyzed showed down-regulation of FUCA-1 expression. Since we [34] and others [35, 36] reported that FUCA-1 is a target of p53 and since the majority of ATCs were reported to harbor *p53* mutations [37], it is highly probable that the lower expression of FUCA-1 in ATCs is related to alterations of the *p53* gene. It must be underlined that some ATC cell lines analyzed, not harboring a p53 mutation, did not show down-regulation of FUCA-1 and expressed normal levels of the protein (results not shown).

We showed here that aggressive anaplastic 8505C thyroid cancer cells adhered well to the human endothelial cells, HUVEC. The extracellular treatment of 8505C cells with bovine α-L-fucosidase, attenuated the adhesion to HUVEC. Since the adhesion of 8505C cells to E-selectin was also strongly inhibited by the extracellular bovine α-L-fucosidase treatment (see Figure 4A), it is likely that the adhesion is mediated by binding fucose-containing structures on 8505C cell surface to E-selectin expressed on the HUVEC cell surface.

Similar results have been reported by Yuan and colleagues (18 and 38), who studied the potential effects of de-fucosylation of the breast cancer cell line, MDA-MB-231, by the same enzyme treatment. In addition, these Authors presented a proof-of principle for the validity of this strategy to inhibit tumor cell invasion: α-L- fucosidase pretreatment significantly decreased the invasive capability of MDA-MB-231 cells *in vivo*. Like leukocytes, cancer cells exit blood vessels by adhering and penetrating through the endothelium [39]. It has been demonstrated that treatment with α-L-fucosidase-1 *in vitro* regulates the migration of leukocytes, thereby significantly reducing the migration of monocytic cells and their adhesion to E- and P-selectin types endothelial receptors [40]. These results suggested that FUCA-1 may play a role in the late stages of inflammation [40] and reinforce the hypothesis of the existence of similar molecular mechanisms that regulate the transport and migration of blood cells into inflammatory foci and the transport and migration of cancer cells endowed with metastatic properties.

In order to better define the role of *FUCA-1* in thyroid carcinogenesis, we stably transfected 8505C cells with *α-L-fucosidase* DNA. The transfected cells had a decreased capacity to adhere to E-selectin and type I collagen, and displayed a decreased migration capacity in wound healing and Matrigel assays and, in addition, a decreased capacity of growth in agar, compared with the same properties of the parental cells. These experiments confirmed the idea that FUCA-1 plays a role in attenuating the aggressive behavior of invasive and metastatic thyroid cancer cells. Furthermore, we performed preliminary experiments to silence the *FUCA-1* gene in the TPC-1 papillary thyroid cancer cell line. TPC-1, which possesses a low *in vitro* invasive potential and is non-tumorigenic *in vivo*, expressed high levels of FUCA-1. The silenced cells acquired a higher invasive potential in Matrigel assays *in vitro* (see Supplementary Figures 2 and 3).

These results strengthen the idea of a potential protective role of α-L-fucosidase in thyroid cancer cells with respect to their invasive *in vitro* capacities.

In order to elucidate how enforced expression of *FUCA-1*, modulated gene expression, we compared the differential expression profiles of the two cell lines: 8505C and *FUCA-1*-transfected 8505C cells. The most differentially expressed genes were those belonging to the Rho/Rac GTPases activating protein (GAPs) and RHO nucleotide exchange factors (GEFs) family of proteins. Activation of Rho/Rac small GTPases has recently been found related to the endothelial transmigration properties of cancer cells [41, 42]. In particular, the Rho GAP genes, which down-regulate Rho GTPase, were dramatically up-regulated in the transfected versus the parental cell line. Conversely, the Rho GEF genes, responsible for maintaining the GTP-bound Rho protein in an active state, were down-regulated in the transfected cells, thus confirming the importance of the activity of the Rho small G protein in driving the aggressive phenotype of 8505C anaplastic cells. Since FUCA-1 expression down-regulates genes involved in pathways related to the epithelial/mesenchymal transition (EMT) such as CXCR4, Rho GEFs, tyrosine kinase receptors, Wnt/β-catenin, Hedgehog, Yap/Taz, and Notch, it is conceivable that loss of FUCA-1 is required in thyroid cancer cells for acquiring an invasive phenotype. A gene expression profile showing the characteristics of fully de-differentiated cells has been found also by van Staveren et al., [43] who have analyzed several PTC and ATC derived cell lines among which also the 8505C cells line. The mechanism by which the transfected *FUCA-1* DNA influences signaling pathways in the 8505C cells remains to be elucidated. It is noteworthy that aberrant glycosylation is causally associated with the acquisition of all the hallmarks in cancer cell signaling, tumor cell dissociation and invasion, cell-matrix interactions, angiogenesis, metastasis and immune modulation (reviewed in 44).

The present results support the evidence that α-L-fucosidase expression is essential to attenuate the malignant and invasive phenotype of thyroid cancer. There is general agreement in considering that protein fucosylation is crucial for leukocyte adhesion to the vasculature, pointing to its role in cell adhesion and motility [45]. The findings presented here support the idea that increased fucosylation contributes to higher invasive properties of both blood and cancer cells. It remains to be clarified how an enzyme whose activity is mainly confined to lysosomes could exert its effects on glycosylated proteins present on the cell membrane. It must be pointed out, in this respect, that, even though α-L-fucosidase-1 is usually found as a soluble component of lysosomes, this enzyme was also found as a membrane fraction- associated in brain [46] and colon adenocarcinoma HT 29 cells [47] and as a plasma membrane-associated in rat spermatozoa [48, 49]. Indeed, by the different approaches used, all the cells tested (hematopietic, epithelial, mesenchimal) were found to express this cell surface protein with α-L-fucosidase activity, which represents 10-20% of the total cellular fucosidase activity and it has been speculated that an alternative traffic pathway for the plasma membrane α-L-fucosidase works on the rapid turnover of glycoproteins [47]. It is conceivable that it is this fraction of the enzyme activity that exerts its action on the membranes of normal thyroid cells and of well differentiated papillary thyroid cancer cells, and contributes to render these cells less fucosylated and therefore less capable of migrating and invading endothelial cells, whereas it is its low expression or absence from less differentiated and more aggressive thyroid papillary and anaplastic cancer cells, which makes them more fucosylated and more capable of adhering to cellular matrix proteins (like collagen type I), to endothelial receptor proteins, such as E-selectin and which makes them more capable of migrating and invading the surrounding tissues. Future studies will be necessary to characterize the levels and the nature of the fucosylated compounds present on the membrane glycoproteins of PTC vs ATC samples and cell lines.

The results presented here open the possibility of new therapeutic strategies for metastatic cancer cells with the use of inhibitors of the interaction between glycoprotein ligands and their receptors on endothelial cells.

It is noteworthy, in this respect, that a study in an animal model of sickle cell crisis was carried out with a synthetic pan-selectin inhibitor (a mimic of sialyl-LewisX) by Chang and colleagues [50] that showed how this small molecule, GMI-1070, which contains a fucose-mimicking portion, effectively competes for the carbohydrate docking domains of E-, L- and P-selectins, inhibiting pro-inflammatory and pro-coagulant responses. Indeed, treatment with GMI-1070 reversed acute vascular occlusions in sickle cell mice [50]. This compound has entered phase 2 clinical trials and its use has been extended to inhibit the invasive properties of cancer cells.

Finally, the studies reported here clearly indicate that the low expression of α-L-fucosidase -1 in thyroid cancer correlates with a higher aggressiveness and metastatic potential of these types of human cancers. It is therefore possible that the low levels of the protein detected by immunohistochemistry or by other means may represent a valuable aid in the prognosis of well-differentiated thyroid cancers having a more aggressive behavior and of anaplastic thyroid cancer, with respect to those having a more favorable prognosis. It is important to notice that similar results of low expression of the enzyme in less differentiated and more aggressive cancers have been found recently also in breast cancer by several studies [51, 52] including one from our laboratory (*In preparation*) and have been related to the worst outcome of breast cancer patients. It is therefore anticipated that the detection of low levels of α-L-FUCA-1 may become a valuable indicator of aggressiveness and clinical outcome not only for thyroid cancer patients but for several human cancers.

## MATERIALS AND METHODS

### Compounds

For *in vitro* experiments, recombinant Human E-selectin/CD63E from R&D (Minneapolis, MN, USA) systems was dissolved in H2O at a concentration of 1mg/ml and stored at −20°C. Collagen type I from BD Biosciences (Heidelberg, Germany) was used at a concentration of 8μg/ml. The α-L-fucosidase solution from bovine kidney was purchased by Sigma (F5884) (Sigma Aldrich, St Louis, MO, USA) and dissolved in PBS at concentration of 1mU/μl. Lewis-Y tetrasaccharide (#H1611), Sialyl Lewis A (#A2512), Lewis X trisaccharide (# B0910), Lewis A trisaccharide (#B0910) and Sialyl Lewis x (#G2212) were purchased from Santa Cruz Biotecnology (Heidelberg, Germany) and dissolved in PBS at a final concentration of 1μg/μl. 4-Methylumbelliferyl α-L-fucopyranoside (4MU) was purchased from Sigma-Aldrich (M8527), dissolved in H_2_O and used at a final concentration of 500μM.

### Immunohistochemistry experiments

Immunohistochemical analysis of FUCA-1 expression was performed on formalin-fixed, paraffin-embedded (FFPE) tumor sections using a rabbit polyclonal anti-FUCA-1 antibody (Proteintech Proteintech Group, Inc. Rosemont, IL, USA). Sections were stained using the Ventana automated slide stainer (Ventana Medical Systems, Tucson, Az, USA) with diluition 1: 100. Expression of FUCA-1 in tumor and normal cells was evaluated independently by two investigators (C.U. and F.B.) who were blinded to clinico-pathological data. Cytoplasmic staining of ≥25% of the tumor cells was considered positive, following the score: 0 <25%; + 25-50%; ++ 50-75%; +++ ≥75%. The possible association of FUCA-1 expression with clinico-pathological features was investigated by statistical analysis

### Human thyroid biopsies

The study included 104 patients (33 with diagnosis of papillary carcinoma (PTC), 26 with poorly differentiated carcinomas (PDTCs) 33 ATCs, and 12 control thyroid tissue samples obtained from neck dissection of patients with non-thyroid carcinomas who underwent total/near-total thyroidectomy at the Department of Surgical, Medical, Molecular Pathology and Critical Area of the University of Pisa, Italy, from 2010 to 2014.

Hematoxylin-eosin stained sections of patients from the archives of the section of Pathology of the University of Pisa were re-evaluated independently by two pathologists (C.U., F.B.). A diagnostic concordance rate of 98% was achieved between the two investigators. Rare discordant cases were eliminated. Tumors were re-classified according to the WHO 2004 histopathological criteria. For all cases, clinico-pathological data were investigated such as age, tumor size, histotype, absence of tumor capsule, presence of extrathyroid infiltration, multifocality, lymph-node metastasis. This retrospective work was conducted anonymously and it conforms to the principles of the Helsinki Declaration of 1975. Informed consent was achieved one day before surgery together with the surgical one.

### Cell lines and cell culture conditions

TPC1 cells were originally obtained by M. Nagao (Carcinogenesis Division, National Cancer Center Research Institute, Tokyo, Japan). BCPAP were obtained by the primary source (N. Fabien, CNRS URA 1454, University of Medecine Lyon-Sud, Oullins, France). 8505C and CAL62 anaplastic carcinoma cells were purchased from DSMZ (Deutsche Sammlung von Mikroorganismen und Zellkulturen GmbH, Braunschweig, Germany). All cells were grown in either Dubecco's modified MEM (DMEM) or RPMI1640 medium supplemented with 10% fetal bovine serum (FBS). All media were supplemented with 2 mM L- glutamine and 100 units/ml penicillin-streptomycin (GIBCO). HUVEC cells were kindly provided by Prof. N. Montuori (University of Naples Federico II, Naples, Italy) and grown in endothelial cell basal medium (EBM®) from Lonza (Basel, Switzerland) (#CC-3121) supplemented with bovine brain extract (BBE) (#CC-4092C), ascorbic acid (#CC-4116C), gentamycin sulfate amphotericin-B (GA-1000) (#CC-4081C), recombinant human epidermal growth factor in a buffered BSA saline solution (rhEGF) (#CC-4017C), hydrocortisone (#CC-4035C), and FBS (CC-4101C). Cell lines from fucosidosis patients were kindly provided by Gaslini Biobank, Bioresource, Genoa, Italy [53].

### Cell proliferation assay

2×105 cells were plated in 60-mm dishes. Cells were kept in RPMI 1640 supplemented with 10% FBS. Cells were counted in triplicates every 24h.

### α-L-fucosidase assay

Cells cultured in standard conditions were collected in 100μl of H_2_O and centrifuged at 2.500 rpm for 3 min. Cell pellets were resuspended in 100μl of α-L-FUCA1 buffer pH 5.5 containing 100mM Na Citrate, 30 mM NaCl, 5 mM Mg Cl 2, 1 mM DTT, and 0.02% Na N3. Cells were sonicated for 40” (Misonix Sonicator 3000). Cell lysates (100 μl) were assayed in α-L-FUCA1 buffer, pH 5.5, containing 0.5 mM 4-methyl-umbellypheryl-α-fucopyranoside, (4-MU), as substrate. The enzyme activity was measured at 37°C by following the releasing of 4-MU (excitation 360 nm, emission 449 nm). One enzymatic unit was defined as the amount of the enzyme that produced 1 pmol of 4-MU per 1 min.

### GFP labeling of 8505C cells

The GFP plasmid (pcDNA3-EGFP) was a generous gift of Dr. Mario Chiariello (IEOS, Naples, Italy). 8505C cells (4.000.000/0.1ml of DMEM containing 4μg plasmid DNA) were transfected by electroporation (pulse voltage :900, pulse width :30, pulse number: 2). 48 hours after the cells were diluted 1:15 with DMEM with 10% FBS and thereafter the cells were cultured in the presence of G418. The 8505C-GFP cells thus isolated were kindly provided by Prof. Rosa Marina Melillo, University of Naples Federico II, Naples, Italy.

### Transfection of 8505C cells with *FUCA-1*

For transfection assay, the GFP-tagged ORF clone of Homo sapiens *alpha-L-fucosidase-1*, (tissue FUCA1) as transfection-ready DNA (#PS100019) from OriGene Technologies was used. Cells were transfected with 5μg *alpha-L-FUCA1* plasmid using FuGENE® HD Transfection Reagent (Promega) following manufacturer's instructions. The day before transfection, cells were plated in 35-mm dishes at 40% of confluence in RPMI 1640 supplemented with 10% FBS. After 72 h Geneticin® (GIBCO by Life Technologies, Thermo Fischer Scientific, Waltham, MA, USA) added at final concentration of 1.25mg/ml was used to select Geneticin–resistant mass population.

### Static adhesion assay to immobilized substrates

96 wells multiwell plates (not for cell culture, Falcon 3915) were treated with either 100 μl/ well of a solution of E-selectin (2μg/ml) diluted in TBS-Tween with 2mMoles/L of CaCl_2_, of fibronectin (5μg/ml) dissolved in 5mMoles/L MgCl_2_ or of Collagen type I (8μg/ml). Some wells were left either untreated or filled with 100μL/well of 1%BSA, to determine the unspecific binding. The multiwell plates were left overnight at 4°C and covered with parafilm. The next day the supernatants were carefully aspirated, each well was washed twice with 100μl/well of PBS and then blocking was performed with 100μl blocking solutions (1% BSA + TBS-Tween in 2mMoles/ L of CaCl_2_ for E-selectin and 1% BSA + 5mMCaCl_2_ for fibronectin or collagen/well), for 1h at 37°C. After blocking and aspiration of the blocking solution each well was washed with 100μl PBS. The cells were added (either 20,000, 50,000 or 100,000/ well suspended in 100μl of DMEM or RPMI medium) and incubated for 3 h at 37°C. After the incubation the supernatants were aspirated and each well was washed twice with 100μl/well PBS, then 100μl/ well of 11% gluteraldehyde were added and left overnight at room temperature under a chemical hood. The next day the gluteraldehyde was aspirated and each well washed twice with 100μl PBS. The cells were stained with 100μl/well of 0.1% crystal violet for 15 min at room temperature. The staining solution was carefully aspirated, each well was washed twice with 100μl/well H2O and the plates inverted on filter paper for a few minutes to drain the excess stain and water. Absorbance at 595nm was measured with an ELISA reader and the plate photographed [39, 54]. Inhibition of adhesion by the treatment with bovine a-L-fucosidasewas measured as described [55]. Briefly, for the treatment with bovine α-L-fucosidase, the 8505C cells were divided in 4 aliquots in 4 different 10 ml Falcon tubes (each containing 1x 10^6^ cells/ml). One aliquot was untreated, the other three aliquots were treated with three different final concentrations of bovine α-L-fucosidase (6.7,13.3 and 67 mU/ml). All samples were left for three hours at 37°C under shaking. Following incubation, triplicates of each sample (1.000.000 cells in 100μL/ well) were plated on the microtiter plate, as described above and left in the incubator for 3 h.

### Adhesion of cancer cells to HUVEC cells

Glass slides were sterilized by UV and then placed in six multiwall plate (Falcon N° 3046) (4 glass slides/well). Each glass slide was treated with a fibronectin solution of 20μg/ml [56]. 270.000 HUVEC cells were suspended in 2 ml of EMB medium (Lonza), plated on each well and left o/n at 37°C. On the next day HUVEC cells were stimulated with IFNγ (100mU/ml) and TNFα (100 U/ml) for 4 h at 37°C. 8505 GFP cells collected by trypsinization of confluent monolayers, were washed with PBS and suspended in DMEM or RPMI1640 medium in order to obtain two different cell numbers (200,000 and 500,000 cells) to be plated on two different glass slides. The EMB medium was aspirated from the wells in which the glass slides were plated and 8505/GFP cells (in 500μl of medium) were added onto each glass slide and incubated for either 3 hours or o/n at 37°C. After incubation the supernatants were removed and the cells fixed with cold 4% paraformaldehyde for 20 minutes. The glass slides were washed once with PBS and then left at 4°C covered with aluminum foil o/n. The subsequent day the PBS was removed and the cells stained with 500μl Hoechst's solution (1:10,000 in PBS) per each glass slide and incubated for 15-20 minutes at room temperature. Each glass slide was then mounted onto a mounting slide with the aid of 5-10μl of mounting solution. After a few minutes of drying the number of adherent green fluorescent protein (GFP)-transfected cells was determined by counting fluorescent cells in several fields with the aid of a fluorescent microscope (Axioscop 2, Zeiss, Oberkochen, Germany). Each glass slide was also photographed and the adhering fluorescent cells counted with the aid of a computer.

For the α-L-fucosidase treatment the 8505 GFP cells were divided, before plating them onto the glass slides in 4 aliquots of 1.0 ml each containing 1,000,000 cells /ml. The first aliquot was not treated (control), the others were treated with final concentrations of 6.7 and 13.3 mU/ml of bovine α-L-fucosidase-1, respectively. The 3 aliquots were incubated under stirring for either 1 hour or 3 hours at 37°C. Inhibition adherence of 8505C cells to HUVEC monolayers that had been plated on the glass slides was measured.

Treatment of 8505C cells with Lewis antigens were performed similarly to the treatment with bovine α-L-fucosidase. Briefly, the GFP-labeled 8505C cells were suspended in DMEM or RPMI at a concentration of 500.000 cells /ml. Ten aliquots of 1.0 ml each were treated with two different concentrations (10 or 100 μg/ml) of Lewis Y, Lewis A, Lewis X, sialyl-Lewis X antigens. The cell suspensions were then directly plated on the glass slides containing the HUVEC monolayer and left to incubate for 3 hours at 37°. The adherent cells were counted as described above.

### Wound healing assay

TPC-1 and 8505C cells were seeded on 35 mm^2^ dishes at 3 × 10^6^ cells/dish, and allowed to grow to confluence. Wound was made by a sterile 10 μl tip, followed by washing out the detached cells. Images were captured at 0, 24 and 36 h by a microscope. Wound healing was evaluated from the area remained empty either at 24 or 36 h.

### Matrigel invasion assay

*In vitro* invasiveness through Matrigel was assayed using transwell cell culture chambers. Briefly, confluent cell monolayers were harvested with trypsin/EDTA and centrifuged at 800xg for 10 minutes. The cell suspension (2×10^5^cells/well) was added to the upper chamber of transwells on pre-hydrated polycarbonate membrane filter of 8 μm pore size (Costar) coated with 35μg Matrigel (Collaborative Research Inc., Washington DC, USA). The lower chamber was filled with complete medium. Culture dishes were incubated at 37°C in 5% CO_2_ and 95% air for 48 hours. Non-migrating cells on the upper side of the filter were wiped off and migrating cells on the reverse side of the filter were stained with 0.1% crystal violet in 20% methanol for 15 minutes, counted and photographed.

### Matrigel 3D assay

*In vitro* invasiveness through Matrigel 3D was assayed using Nunc™ Lab-Tek™ Chambered Coverglass #155383 (Thermo Scientific™). One hundred fifty microliters BD Matrigel Matrix Growth Factor Reduced (GFR) #356230 (BD Biosciences, Heidelberg, Germany) were plated for each chambered coverglass and incubated 30 minutes at 37°C. The cell suspension (5×10^4^ cells/chambered coverglass) was enriched with 2% Matrigel Matrix (GFR) and added to the chamber. Complete medium was added every four days. After fifteen days cells were photographed.

### Soft agar assay

Petri dishes of 60 mm diameter were prepared by adding 7 ml of complete medium containing 0.5% soft agar. 8505C and 8505C-α-L-FUCA1 cells cultured in standard conditions were trypsinized, centrifuged and resuspended in complete medium and medium containing 0.5% soft agar at a ratio 1:3. The cells (5×104/plate) were then overlayed onto the Petri dishes containing the solidified agar medium (1.5 ml/dish) and incubated at 37°C and 5% CO2. Control and transfected cultures were observed under a microscope just after plating, to verify the absence of cell aggregates, and then periodically checked for colonies formation. After 4 weeks, colonies were counted with an optical microscope at a 10X magnification.

### Quantitative real-time PCR

Total RNA was isolated with the RNeasy Kit (Qiagen, Crawley, West Sussex, UK). One μg of RNA from each sample was reverse-transcribed with the QuantiTect® Reverse Transcription kit (Qiagen). PCR reactions were performed in triplicates and fold changes were calculated with the formula: 2- (sample 1 ΔCt - sample 2 ΔCt), where ΔCt is the difference between the amplification fluorescent thresholds of the mRNA of interest and the mRNA of β Actin used as an internal reference.

### Immunoblotting

Protein lysates were prepared according to procedures already described [57]. Briefly, growing cells on the plate were harvested in JS lysis buffer containing 50 mM N-2- hydroxyethylpiperazine-N’-2-ethanesulfonic acid (HEPES; pH 7.5), 1% (vol/vol) Triton X-100, 150 mM NaCl, 5 mM EGTA, 50 mM NaF, 20 mM sodium pyrophosphate, 1 mM sodium vanadate, 2 mM phenylmethylsulphonyl fluoride (PMSF) and 1 μg/ml aprotinin and clarified by centrifugation at 10,000 x*g*. Protein concentration was estimated with a modified Bradford assay (Bio-Rad) and 30 micrograms of lysates were subjected to Western blotting on 12% acrylamide gels. Membranes were probed with the indicated antibodies. Immune complexes were revealed by an enhanced chemiluminescence detection kit (ECL, Amersham Pharmacia Biotech, Piscataway, NJ, USA). Signal intensity was quantified with the Phosphorimager (Typhoon 8600, Amersham Pharmacia Biotech) interfaced with the Image Quant software.

### Antibodies

Anti α-L-fucosidase (FUCA1) is a polyclonal antibody from Proteintech Group (Rosemont, IL, USA) (16420-1-AP) which recognizes the α-L-fucosidase-1 protein. Anti-turbo-GFP-tag is a monoclonal antibody from Origene Technologies (TA150041-100) which recognizes over expressed recombinant proteins containing the turbo-GFP tag fused to either the amino- or carboxy-termini of targeted proteins. Monoclonal anti-α-tubulin (#T9026) was from Sigma Aldrich (St Louis, MO, USA). Secondary antibodies coupled to horseradish peroxidase were from Amersham Pharmacia Biotech (Piscataway, NJ, USA). Monoclonal anti-α-tubulin (#T9026) was from Sigma Aldrich (St Louis, MO, USA). Secondary antibodies coupled to horseradish peroxidase were from Amersham Pharmacia Biotech (Piscataway, NJ, USA).

### Gene expression profiling

The gene expression of the two cell lines (8505C and 8505C/FUCA-1) were analyzed by the microarray Human Gene Expression 8×60k, containing oligonucleotide probes of 60mer, corresponding to the whole human genome. Four different chips (arrays) were hybridized with cDNAs from the two different cell lines with two different fluorofors Cy3 and Cy5. Data obtained from the hybridization have been analyzed to determine the genes differentially expressed in the presence of expression of the FUCA-1 gene and in order to evaluate the statistical significance. The identified genes have been subdivided according to the metabolic pathways in which they are involved.

### TPC-1 cells stably expressing FUCA-1-specific shRNA

FUCA-1-specific shRNA expression vector (#TG312904) and a scrambled control vector (#TR30013) were purchased from OriGene Technologies, (Inc.9620 Medical Center Drive, Suite 200 Rockville). Experimental procedures were done as follows: the day before transfection, cells were plated in 35-mm dishes at 40% of confluence in DMEM supplemented with 10% FBS without antibiotics. Two μg of sh-FUCA-1 and control vector were transfected using FuGENE HD transfection (Promega) reagent according to manufacturer's instructions. Seventy-two hours after transfection, culture medium was supplemented with puromycin (Sigma-Aldrich) at final concentration of 1μg/μl for 14 days. Stably transfected cells were screened by RT-PCR as previously described.

### Statistical analysis

Unpaired Student's t tests using the Instat software program (Graphpad Software Inc) were performed to compare cell growth. All P values were two- sided, and differences were considered statistically significant at P <0.05. The χ^2^ test and Mann-Whitney U test were used to analyze the correlation of clinical and pathological data with FUCA-1 expression. The Statistica 6.0 software program (StatSoft, Inc. Tulsa, OK) was used. P values <0.05 were considered statistically significant.

## SUPPLEMENTARY FIGURES AND TABLES




