# Supplementary material for: Human α-L-fucosidase-1 attenuates the invasive properties of thyroid cancer
Source: Oncotarget. 2017 Feb 23;8(16):27075–92. doi: 10.18632/oncotarget.15635 (PMC5432319; doi:10.18632/oncotarget.15635)
Supplement: Supplementary file 1 [file oncotarget-08-27075-s001.pdf]

Human *α-L-fucosidase-1* attenuates the invasive properties of thyroid cancer

SUPPLEMENTARY FIGURES AND TABLES

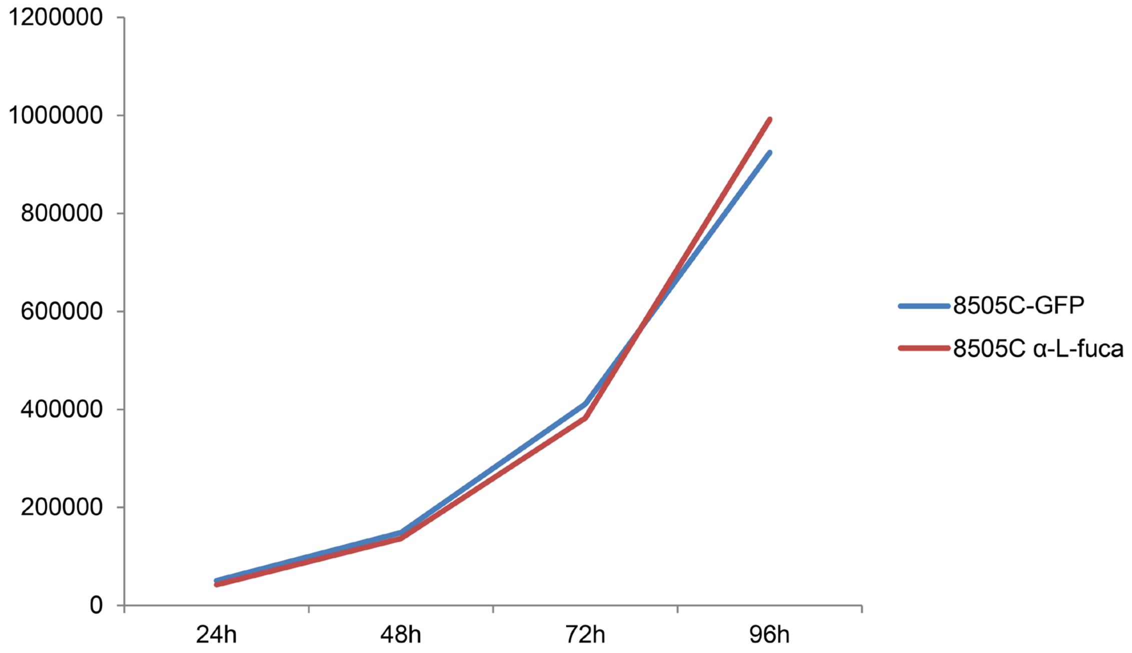

Supplementary Figure 1: Growth curve of stably transfected 8505C-GFP and 8505C α-L-fuca. 50.000 cells were plated and counted every day up to 96 hours (see Materials and Methods). No significant differences were observed between control and α-L-fuca-transfected cells.

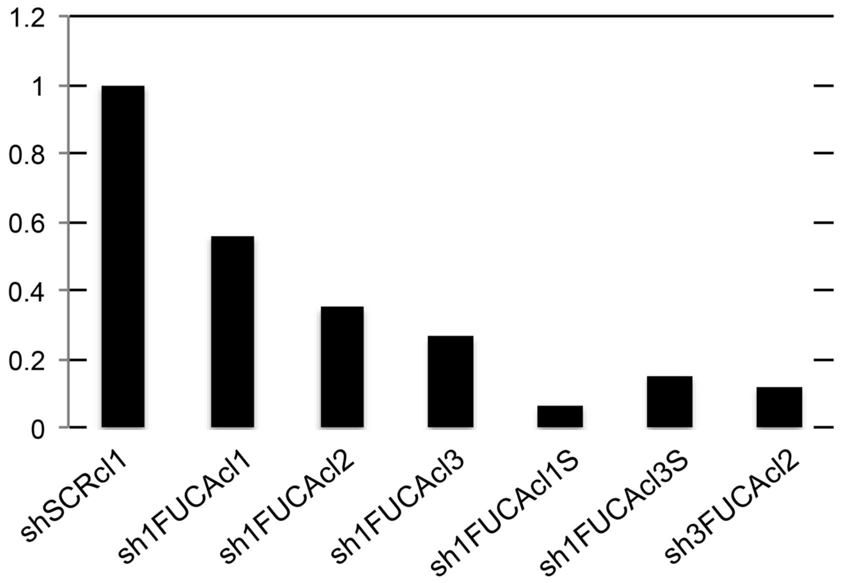

Supplementary Figure 2: RNA extracted from either control scrambled TPC1 (shSCRcl1) or from sh1FUCAcl1, sh1FUCAcl2, sh1FUCAcl3, sh3FUCAcl2, or from the sorted clones sh1FUCAcl1S and sh1FUCAcl3S and fucosidase relative expression (fold reduction) was analyzed by q-RT-PCR.

A

% ADHESION WITH RESPECT TO TPC1shSCR cl1

	TPC1sh1 FUCA cl1	TPC1sh1 FUCA cl3
COLLAGEN I	123%	160%

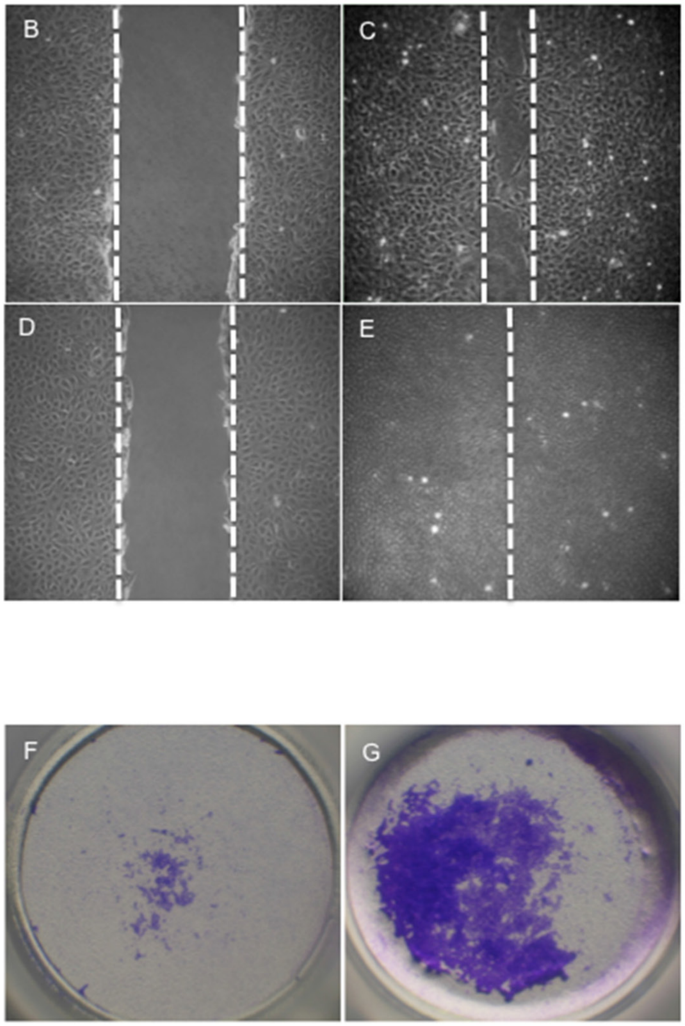

Supplementary Figure 3: A. Adhesion to Collagen type I of TPC1 cell clones silenced for the FUCA1 gene (see Materials and Methods). B-E. Wound healing assay with the two clones, TPC1shSCR cl1 (B and C, 0 time and 36 hours after the wound, respectively) and TPC1sh1FUCA cl3 (D and E, 0 time and 36 hours after the wound, respectively). **F-G.** Matrigel assay. 100.000 cells of TPC1shSCR cl1 (F) and of TPC1sh1FUCA cl3 (G) were plated at time 0 into matrigel canisters and the migration was measured 48 hours after plating (see Materials and Methods).

Supplementary Table 1: Genes down-regulated by FUCA1 expression

See Supplementary File 1

Supplementary Table 2: Genes up-regulated by FUCA1 expression

Fold change	Gene symbol	Gene name
5.2438755	DIO2	deiodinase, iodothyronine, type II
4.2457094	ARHGAP44	Rho GTPase activating protein 44
3.016162	SOD3	superoxide dismutase 3, extracellular
2.7995398	ARHGAP6	Rho GTPase activating protein 6
2.2704682	ARHGAP25	Rho GTPase activating protein 25
1.3379228	ARHGAP42	Rho GTPase activating protein 42
1.1928251	ARHGAP4	Rho GTPase activating protein 4
1.0785917	ARHGAP9	Rho GTPase activating protein 9
1.0102775	BRCA2	breast cancer 2, early onset
