# Supplementary material for: Human α-L-fucosidase-1 attenuates the invasive properties of thyroid cancer
Source: Oncotarget. 2017 Feb 23;8(16):27075–92. doi: 10.18632/oncotarget.15635 (PMC5432319; doi:10.18632/oncotarget.15635)
Supplement: Supplementary file 2 [file oncotarget-08-27075-s002.docx]

**Supplementary information**

**Human α-L-fucosidase-1 attenuates the invasive properties of thyroid cancer**

Giancarlo Vecchio et al.

**Table I**

**Genes down-regulated by FUCA1 expression**

| **Fold change** | **Gene symbol** | **Gene name** |
| --- | --- | --- |
| -8.783249 | SLITRK4 | SLIT and NTRK-like family, member 4 |
| -7.7114964 | GRB10 | growth factor receptor-bound protein 10 |
| -6.9468513 | PRKCZ | protein kinase C, zeta (PRKCZ), transcript variant 1 |
| -4.8102217 | CXCR4 | chemokine (C-X-C motif) receptor 4 |
| -4.500466 | WNT7A | wingless-type, member 7A |
| -4.390707 | ARHGEF5 | Rho guanine nucleotide exchange factor (GEF) 5 |
| -4.2045083 | FGD2 | FYVE, RhoGEF and PH domain containing 2 |
| -4.075833 | HHIP | hedgehog interacting protein |
| -3.9591885 | HRASLS | HRAS-like suppressor |
| -3.4242704 | ARHGEF35 | Rho guanine nucleotide exchange factor (GEF) |
| -2.552536 | IGFBPL1 | insulin-like growth factor binding protein-like 1 |
| -2.5002267 | INSR | insulin receptor (INSR), transcript variant 1 |
| -2.496904 | WNT5B | wingless-type MMTV integration site family, member 5B |
| -2.4367707 | SFRP1 | secreted frizzled-related protein 1 |
| -2.420014 | ARMCX4 | armadillo repeat containing, X-linked 4 |
| -2.294403 | SMO | smoothened, frizzled class receptor |
| -2.1417503 | ARHGEF37 | Rho guanine nucleotide exchange factor (GEF) 37 |
| -2.0483024 | ADAM19 | ADAM metallopeptidase domain 19 |
| -2.0200534 | FZD2 | frizzled class receptor 2 |
| -2.0004148 | MAP3K14 | mitogen-activated protein kinase kinase kinase 14 |
| -1.9638394 | GRAP | GRB2-related adaptor protein |
| -1.8908923 | DKK3 | dickkopf WNT signaling pathway inhibitor 3 |
| -1.8830662 | ADAMTS1 | ADAM metallopeptidase with thrombospondin type 1 motif, 1 |
| -1.8286839 | CCNF | cyclin F |
| -1.7913268 | PRKACB | protein kinase, cAMP-dependent, catalytic, beta |
| -1.7799492 | AXIN2 | axin 2 |
| -1.746341 | PLK1 | polo-like kinase 1 |
| -1.7422855 | CTNNBIP1 | catenin, beta interacting protein 1 |
| -1.7069626 | PLK2 | polo-like kinase 2 |
| -1.7008526 | CCNB1 | cyclin B1 |
| -1.6909592 | NRARP | NOTCH-regulated ankyrin repeat protein |
| -1.6701806 | RASD1 | RAS, dexamethasone-induced 1 |
| -1.6386732 | RAPGEF3 | Rap guanine nucleotide exchange factor (GEF) 3 |
| -1.6279031 | CITED1 | Cbp/p300-interacting transactivator, with Glu/Asp-rich carboxy-terminal domain, 1 |
| -1.6259086 | ARHGEF9 | Cdc42 guanine nucleotide exchange factor (GEF) 9 |
| -1.6215414 | KIT | v-kit Hardy-Zuckerman 4 feline sarcoma viral oncogene homolog |
| -1.5511128 | RAPGEF6 | Rap guanine nucleotide exchange factor (GEF) 6 |
| -1.5097318 | ARHGEF40 | Rho guanine nucleotide exchange factor (GEF) 40 |
| -1.5034473 | RTKN2 | rhotekin 2 |
| -1.4406505 | FUT4 | fucosyltransferase 4 (alpha (1,3) myeloid-specific) |
| -1.4136598 | ADAMTSL1 | ADAMTS-like 1 |
| -1.3724201 | NGEF | neuronal guanine nucleotide exchange factor |
| -1.3535638 | VEGFA | vascular endothelial growth factor A |
| -1.3504982 | TRIO | trio Rho guanine nucleotide exchange factor |
| -1.3457074 | AURKA | aurora kinase A |
| -1.1170487 | FGFR3 | fibroblast growth factor receptor 3 |
| -1.0885444 | THRA | thyroid hormone receptor, alpha |
| -1.0676752 | RHOT1 | ras homolog family member T1 |
| -1.0652103 | HRAS | Harvey rat sarcoma viral oncogene homolog |
| -1.029585 | RASAL3 | RAS protein activator like 3 |
| -1.0259818 | ADAMTS5 | ADAM metallopeptidase with thrombospondin type 1 motif, 5 |
| -1.022284 | EREG | epiregulin |
| -1.021966 | MYBL2 | v-myb avian myeloblastosis viral oncogene homolog-like 2 |
| -1.004879 | DIRAS1 | DIRAS family, GTP-binding RAS-like 1 |
